# Supplementary material for: Queueing theory model of mTOR complexes’ impact on Akt-mediated adipocytes response to insulin
Source: PLoS One. 2022 Dec 27;17(12):e0279573. doi: 10.1371/journal.pone.0279573 (PMC9794039; doi:10.1371/journal.pone.0279573)
Supplement: S1 File — Supporting Information file contains values of literature concentrations used in the model and reaction equations and kinetic constants used in the model. The source code is freely available for download at https://github.com/UTP-WTIiE/IrsMtorcQueuesSimulation, implemented in C# supported in Linux or MS Windows. (DOCX) [file pone.0279573.s001.docx]

# Queueing theory model of mTOR complexes’ impact on Akt-mediated cell response to insulin

Sylwester M. Kloska^1,*^, Krzysztof Pałczyński^2^, Tomasz Marciniak^2^, Tomasz Talaśka^2^, Marissa Miller^3^, Beata J. Wysocki^4^, Paul H. Davis^4^, Ghada A. Soliman^5^, and Tadeusz A. Wysocki^2,3^

^1^ Department of Forensic Medicine, Nicolaus Copernicus University Ludwik Rydygier Collegium Medicum, Bydgoszcz, Poland

^2^ Faculty of Telecommunications, Computer Science and Electrical Engineering, University of Science and Technology, Bydgoszcz, Poland

^3^ Department of Electrical and Computer Engineering, University of Nebraska-Lincoln, Omaha, Nebraska, United States of America

^4^ Department of Biology, University of Nebraska at Omaha, Omaha, United States of America

^5^ Department of Environmental, Occupational, and Geospatial Health Sciences, City University of New York, Graduate School of Public Health and Healthy Policy, New York, United States of America

Corresponding author e-mail: [503013@stud.umk.pl](mailto:503013@stud.umk.pl)

Table 1 Values of literature concentrations used in the model.

| Label | Initial conc. | ± | Units |
| --- | --- | --- | --- |
| I | 1,200 | - | nM |
| IR | 0.0009 | - | nM |
| IR-I | - | - | - |
| pIR-I | - | - | - |
| \|pIR-I\| | - | - | - |
| pIR-II | - | - | - |
| \|pIR-II\| | - | - | - |
| IRS1/3 | 60 | 40 | nM |
| pIRS1/3 | - | - | - |
| PI3K | 200 | 70 | nM |
| pIRS1/3-PI3K | - | - | - |
| pIRS1/3-PI3K* | - | - | - |
| PI3K* | - | - | - |
| PI3K*-PI | - | - | - |
| PI3K*-PIP3 | - | - | - |
| PIP3 | - | - | - |
| PI | 300 | 30 | nM |
| PTEN-PIP3 | - | - | - |
| PTEN-PI | - | - | - |
| PTEN | 50 | 5 | nM |
| pPTEN-PTEN | - | - | - |
| PTEN-PTEN | - | - | - |
| pPTEN | - | - | - |
| Akt | 100 | 3 | nM |
| Akt-PIP3 | - | - | - |
| pAkt-PIP3 | - | - | - |
| PP2A-Akt-PIP3 | - | - | - |
| PP2A-pAkt-PIP3 | - | - | - |
| PP2A-ppAkt-PIP3 | - | - | - |
| PP2A | 10 | 2.5 | nM |
| PP2A-Akt | - | - | - |
| PP2A-pAkt | - | - | - |
| PP2A-ppAkt | - | - | - |
| ppAkt | - | - | - |
| ppAkt-PIP3 | - | - | - |
| PDK | - | - | - |
| As160 | 108 | - | nM |
| pAs160 | 12 | - | nM |
| PP2A-pAs160 | - | - | - |
| PP2A-As160 | - | - | - |
| RabGTP | 0.0965 | - | nM |
| RabGDP | 0.8685 | - | nM |
| RabGTP-As160 | - | - | - |
| GEF | 0.51 | - | nM |
| RabGDP-GEF | - | - | - |
| GLUT4 | 370000 | - | - |
| GLUT4 in vesicles | 20000 | - | - |
| S6K | 60000 | - | pM |
| pS6K | - | - | - |
| pS6K-IRS1/3 | 0.30241 | - | nM |
| mTORC1 | 3960.783 | - | pM |
| pmTORC1 | - | - | - |
| GAPDH-pmTORC1 complex |  |  |  |
| Amino acids | 10199.42 | - | pM |
| AMPK | 6709 | - | pM |
| pAMPK | - | - | - |
| mTORC2 | 634.15 | - | pM |
| pmTORC2 | - | - | - |
| TSC1_TSC2 | 4880.49 | - | pM |
| p(TSC1_TSC2) | - | - | - |
| PDK2 | 1235.91 | - | pM |
| pPDK2 | - | - | - |

Table 2 Reaction equations and kinetic constants used in the model.

| Reaction equation | Kinetic constants | | ± | Source |
| --- | --- | --- | --- | --- |
| $V_{0}=k_{0}*[I]*[IR]-(k-0)*[IR-I]$ | $k_{0}$  $k-0$ | 0.06  0.2 | -  - | [1] |
| $V_{1}=k_{1}*[IR-I]$ | $k_{1}$ | 2,500 | - | [1] |
| $V_{2}=k_{2}*[pIR-I]$ | $k_{2}$ | 0.2 | - | [1] |
| $V_{3}=k_{3}*[pIR-I]-(k-3)*[\vert pIR-I\vert]$ | $k_{3}$  $k-3$ | 0.0021  0.00021 | -  - | [1] |
| $V_{4}=k_{4}*[I]*[pIR-I]-(k-4)*[pIR-II]$ | $k_{4}$  $k-4$ | 0.06  20 | -  - | [1] |
| $V_{5}=k_{5}*[pIR-II]-(k-5)*[\vert pIR-II\vert]$ | $k_{5}$  $k-5$ | 0.0021  0.00021 | -  - | [1] |
| $V_{6}=\frac{k_{6}*\{[IRS1/3]([pIR-I]+[pIR-II])\}}{[IR]-(k-6)*[pIRS1/3]}$ | $k_{6}$  $k-6$ | 4.16  1.4 | -  - | [1] |
| $V_{7}=k_{7}*(\left[ {pIRS1}/3 \right]*\left[ PI3K \right]-k_{d,7}*\left[ {pIRS1}/3-PI3K \right])$ | $k_{7}$  $k_{d,7}$ | 3  1 | 1  - | [1,2] |
| $V_{8}=k_{8}*\left[ {pIRS1}/3-PI3K \right]-\left( k-8 \right)*[{pIRS1}/3-PI3K*]$ | $k_{8}$  $k-8$ | 300  0 | 30  - | [1,2] |
| $V_{9}=k_{9}*\left[ {pIRS1}/3-PI3K* \right]-\left( k-9 \right)*[{pIRS1}/3*PI3K*]$ | $k_{9}$  $k-9$ | 13,500  0 | -  - | [2,3] |
| $V_{10}=k_{10}*[PI3K*]$ | $k_{10}$ | 900 | 130 | [2,3] |
| $V_{11}=k_{11}*(\left[ PI \right]*\left[ PI3K* \right]-k_{d,11}*\left[ PI3K*-PI \right])$ | $k_{11}$  $k_{d,11}$ | 0.03  140 | 0.006  - | [2,3] |
| $V_{12}=k_{12}*[PI3K*-PI]$ | $k_{12}$ | 30 | - | [2,3] |
| $V_{13}=k_{13}*[PI3K*-PIP3]$ | $k_{13}$ | 30 | - | [3] |
| $V_{14}=k_{14}*(\left[ PIP3 \right]*\left[ PTEN \right]-k_{d,14}*\left[ PTEN-PIP3 \right])$ | $k_{14}$  $k_{d,14}$ | 8,000  0.01 | -  0.004 | [2,3] |
| $V_{15}=k_{15}*[PTEN-PIP3]$ | $k_{15}$ | 15 | 5 | [3] |
| $V_{16}=k_{16}*[PTEN-PI]$ | $k_{16}$ | 3.6 | 1 | [3] |
| $V_{17}=\frac{V_{max,17}*[PTEN]}{k_{m,17}+\left[ PTEN \right]}$ | $V_{max,17}$  $k_{m,17}$ | 150  2 | 50  - | [3] |
| $V_{18}=k_{18}*(\left[ PTEN \right]*\left[ pPTEN \right]-k_{d,18}*[pPTEN-PTEN]$ | $k_{18}$  $k_{d,18}$ | 1  2.2 | 0.4  - | [3] |
| $V_{19}=k_{cat,19}*[pPTEN-PTEN]$ | $k_{cat,19}$ | 150 | - | [3] |
| $V_{20}=k_{20}*[PTEN-PTEN]$ | $k_{20}$ | 150 | - | [3] |
| $V_{21}=k_{21}*(\left[ PIP3 \right]*\left[ Akt \right]-k_{d,21}*\left[ Akt-PIP3 \right])$ | $k_{21}$  $k_{d,21}$ | 15,000  20 | -  6 | [2,3] |
| $V_{22}=\frac{V_{max,22}*[Akt-PIP3]}{k_{m,22}+[Akt-PIP3]}$ | $V_{max,22}$  $k_{m,22}$ | 15,000  0.1 | 5,000  0.035 | [2,3] |
| $V_{23}=\frac{V_{max,23}*[pAkt-PIP3]}{k_{m,23}+[pAkt-PIP3]}$ | $V_{max,23}$  $k_{m,23}$ | 15,000  0.1 | 5,000  0.035 | [2,3] |
| $V_{24}=k_{24}*(\left[ ppAkt-PIP3 \right]*\left[ PP2A \right]-k_{d,24}*\left[ PP2A-ppAkt-PIP3 \right])$ | $k_{24}$  $k_{d,24}$ | 3  0.1 | -  - | [3] |
| $V_{25}=k_{25}*[PP2A-ppAkt-PIP3]$ | $k_{25}$ | 45 | 20 | [3] |
| $V_{26}=k_{26}*\left[ pAkt-PIP3 \right]*[PP2A]$ | $k_{26}$ | 3 | - | [3] |
| $V_{27}=k_{27}*[PP2A-pAkt-PIP3]$ | $k_{27}$ | 0.3 | - | [3] |
| $V_{28}=k_{28}*[PP2A-pAkt-PIP3]$ | $k_{28}$ | 45 | 20 | [3] |
| $V_{29}=k_{29}*[PP2A-Akt-PIP3]$ | $k_{29}$ | 30 | - | [3] |
| $V_{30}=k_{30}*[ppAkt-PIP3]$ | $k_{30}$ | 30 | - | [3] |
| $V_{31}=k_{31}*(\left[ ppAkt \right]*\left[ PP2A \right]-k_{d,31}*\left[ PP2A-ppAkt \right])$ | $k_{31}$  $k_{d,31}$ | 3  0.1 | -  - | [3] |
| $V_{32}=k_{32}*[PP2A-ppAkt]$ | $k_{32}$ | 45 | 20 | [3] |
| $V_{33}=k_{33}*[PP2A-pAkt]$ | $k_{33}$ | 45 | 20 | [3] |
| $V_{34}=k_{34}*[PP2A-Akt]$ | $k_{34}$ | 30 | - | [3] |
| $V_{35}=\frac{V_{max,35}*[As160]}{k_{m,35}+[As160]}$ | $V_{max,35}$  $k_{m,35}$ | 0.00003288  24,810 | -  - | [4] |
| $V_{36}=k_{36}*(\left[ pAs160 \right]*\left[ PP2A \right]-k_{d,36}*\left[ PP2A-pAs160 \right])$ | $k_{36}$  $k_{d,36}$ | 3  0.1 | -  - | [3] |
| $V_{37}=k_{37}*[PP2A-pAs160]$ | $k_{37}$ | 45 | 20 | [3] |
| $V_{38}=k_{38}*[PP2A-As160]$ | $k_{38}$ | 30 | - | [3] |
| $V_{39}=\frac{(k_{off,As160}+k_{cat,As160})}{\frac{k_{M,As160}}{D}*\left[ RabGTP \right]*[As160]}$ | $k_{off,As160}$  $k_{cat,As160}$  $k_{M,As160}$  $D$ | 600  324  230  250 | -  -  3  - | [5] |
| $V_{40}=k_{off,GEF}*[RabGTP-As160]$ | $k_{off,GEF}$ | 600 | - | [5] |
| $V_{41}=k_{cat,GEF}*[RabGTP-As160]$ | $k_{cat,GEF}$ | 234 | - | [5] |
| $V_{42}=\frac{(k_{off,GEF}+k_{cat,GEF})}{\frac{k_{M,GEF}}{D}*\left[ RabGDP \right]*[GEF]}$ | $k_{off,GEF}$  $k_{cat,GEF}$  $k_{M,GEF}$  $D$ | 600  234  3,860  250 | -  -  3  - | [5] |
| $V_{43}=k_{off,GEF}*[RabGDP-GEF]$ | $k_{off,GEF}$ | 600 | - | [5] |
| $V_{44}=k_{cat,GEF}*[RabGDP-GEF]$ | $k_{cat,GEF}$ | 234 | - | [5] |
| $V_{45}=k_{45}*[RabGTP]$ | $k_{45}$ | 0.0059 | - | [3,6] |
| $V_{46}=k_{46}*\left[ Glut_{Membrane} \right]*[As160]$ | $k_{46}$ | 1.30E-07 | - | [3,6] |
| $V_{47}=\frac{k_{47}*[Glut_{Vascularized}]}{[As160]}$ | $k_{47}$ | 2.4167E-5 | - | [3,6] |
| $V_{48}=k_{48}*\left[ Amino\_Acids \right]*[mTORC1]$ | $k_{48}$ | 0.0513784 | - | [7] |
| $V_{49}=k_{49}*\left[ pTSC1\_TSC2 \right]*[pmTORC1]$ | $k_{49}$ | 0.999989 | - | [7] |
| $V_{50}=k_{50}*\left[ pmTORC1 \right]*[S6K]$ | $k_{50}$ | 0.00573896 | - | [7] |
| $V_{51}=k_{51}*[pS6K]$ | $k_{51}$ | 0.00528455 | - | [7] |
| $V_{52}=k_{52}*\left[ pTSC1\_TSC2 \right]$ | $k_{52}$ | 0.000100001 | - | [7] |
| $V_{53}=k_{53}*\left[ pAMPK \right]*[{pIRS1}/3]$ | $k_{53}$ | 9.79766 | - | [7] |
| $V_{54}=k_{54}*\left[ AMPK \right]$ | $k_{54}$ | 0.0107215 | - | [7] |
| $V_{55}=k_{55}*\left[ pAMPK \right]*[TSC1\_TSC2]$ | $k_{55}$ | 0.036559 | - | [7] |
| $V_{56}=k_{56}*\left[ ppAkt-PIP3 \right]$ | $k_{56}$ | 7.52842 | - | [7] |
| $V_{57}=k_{57}*\left[ pmTORC2 \right]$ | $k_{57}$ | 0.0174149 | - | [7] |
| $V_{58}=k_{58}*\left[ mTORC2 \right]*[PI3K*]$ | $k_{58}$ | 0.0781585 | - | [7] |
| $V_{59}=k_{59}*[pPDK2]$ | $k_{59}$ | 1 | - | [7] |
| $V_{60}=k_{60}*\left[ pIR-II \right]*[PDK2]$ | $k_{60}$ | 0.1 | - | [7] |
| $V_{61}=k_{61}*\left[ pS6K \right]*[IRS1/3]$ | $k_{61}$ | 1 | - | [7] |
| $V_{62k1}=\frac{k_{c[0]}}{{(stateQ+k}_{c[1]})}$  $V_{62k2}=k_{c[2]}$ | $k_{c\left[ 0 \right]}$  $k_{c[1]}$  $k_{c[2]}$  $stateQ$ | 6394.615666816595  2.3861985982327236  0.54373973880069393  0.00194 | -  -  -  - | estimation based on [7] |
| $V_{62}=V_{62k1}*\left( GAPDH \right)*\left[ pmTORC1 \right]-V_{62k2}*mTORC1 complex$ | $V_{62k1}$ | 2677.6568460259195 | - | estimation based on [7] |
| $V_{63}=V_{62k2}*mTORC1 complex-V_{62k1}*GAPDH*pmTORC1$ | $V_{62k2}$ | 0.54373973880069393 | - | estimation based on [7] |
| $V_{64}=k_{64}*\left[ pS6K \right]*[IRS1/3]$ | $k_{64}$ | 0.0001 | - | [7] |

$k_{i}={nM}^{-1}{min}^{-1};k_{d,i},k_{M,i},k_{M,As160},$ and $k_{M,GEF}=nM;$

$V={nM}/{min};k_{off,As160},k_{cat,As160},k_{off,GEF},$ and $k_{cat,GEF}={min}^{-1};D=$ no units.

References:

1. Sedaghat AR, Sherman A, Quon MJ. A mathematical model of metabolic insulin signaling pathways. Am J Physiol - Endocrinol Metab. 2002;283(5 46-5):1084–101.

2. Hatakeyama M, Kimura S, Naka T, Kawasaki T, Yumoto N, Ichikawa M, et al. A computational model on the modulation of mitogen-activated protein kinase (MAPK) and Akt pathways in heregulin-induced ErbB signalling. Biochem J. 2003;373(2):451–63.

3. Faratian D, Goltsov A, Lebedeva G, Sorokin A, Moodie S, Mullen P, et al. Systems biology reveals new strategies for personalizing cancer medicine and confirms the role of PTEN in resistance to trastuzumab. Cancer Res. 2009;69(16):6713–20.

4. Shin I, Edl J, Biswas S, Lin PC, Mernaugh R, Arteaga CL. Proapoptotic activity of cell-permeable anti-Akt single-chain antibodies. Cancer Res. 2005;65(7):2815–24.

5. Legewie S, Sers C, Herzel H. Kinetic mechanisms for overexpression insensitivity and oncogene cooperation. FEBS Lett [Internet]. 2009;583(1):93–6. Available from: http://dx.doi.org/10.1016/j.febslet.2008.11.027

6. Kozka IJ, Clark AE, Reckless JPD, Cushman SW, Gould GW, Holman GD. The effects of insulin on the level and activity of the GLUT4 present in human adipose cells. Diabetologia. 1995;38(6):661–6.

7. Sonntag AG, Dalle Pezze P, Shanley DP, Thedieck K. A modelling-experimental approach reveals insulin receptor substrate (IRS)-dependent regulation of adenosine monosphosphate-dependent kinase (AMPK) by insulin. FEBS J. 2012;279(18):3314–28.
