# Supplementary material for: Queueing theory model of mTOR complexes’ impact on Akt-mediated adipocytes response to insulin
Source: PLoS One. 2022 Dec 27;17(12):e0279573. doi: 10.1371/journal.pone.0279573 (PMC9794039; doi:10.1371/journal.pone.0279573)
Supplement: Attachment — Submitted filename: Response to Reviewers.docx [file pone.0279573.s002.docx]

**Dear Dr Irina U Agoulnik**,

Thank you for giving us the opportunity to submit a revised draft of our manuscript titled “Queueing theory model of mTOR complexes’ impact on Akt-mediated cell response to insulin” to *PLOS ONE*. We appreciate the time and effort that you and the reviewers have dedicated to providing your valuable feedback on my manuscript. We are grateful to the reviewers for their insightful comments on my paper. We have been able to incorporate changes to reflect most of the suggestions provided by the reviewers. We have highlighted the changes within the manuscript.

Here is a point-by-point response to the reviewers’ comments and concerns.

**Comments from the Editor**

*Even after downloading a high resolution of figure 1, it is impossible to read and understand the flow of the signaling.*

Response: Thank you for pointing this out. We agree with your point and we refined mentioned figure in order to improve its quality and readability.

*Please also clearly describe, which tissues/cells you are modeling for and why, as the insulin receptor signaling is vastly different in WAT, BAT, liver, muscle, adipose, pancreatic and other cells.*

Response: Thank you for pointing this out. As suggested, we have added an explanation that presented model refers to human adipocytes (lines 118-119).

*As suggested by the reviewer, please clearly separate introductory background description from the original research performed for this manuscript. Please provide additional clarifications and descriptions, requested by the reviewer.*

Response: Thank you for these suggestions. We have adapted our work to the guidance and feedback provided in the review.

**Comments from Reviewer 1**

- Comment 1: *The authors need to do more to showcase the previous work (not just reference it), while explaining how the present manuscript builds on/is different from what they (and others?) have already published. The authors may choose to present the work as a validation of already-available information via other methods that could potentially yield novel information, but it is important to be clear on what the current goal is, how much of the goal has been achieved in this manuscript and what future perspective the authors envision. However, the authors should consider defining the theory itself, and the hypothesis upon which its viability is predicated.*

Response: Thank you for pointing this out. We agree with this comment. Therefore, we have added the following comments in the Introduction section: “The research hypothesis of this work is the ability to simulate the cellular response to insulin and track changes in the concentrations of proteins involved in this response using queueing theory based simulation model. The presented model shows the mechanism of mTORC1 influence on mobilization of GLUT4 particles. Since mTORC1 has been reported in literature as having an impact on glucose uptake (20,21).”

“Since then the mTORC1 signaling pathway connections with Akt-mediated insulin response has been described (22,23). This work presents a model where those connections have been included.”.

- Comment 2: *In the methodology and result sections, it is important to be clear about where the authors are showcasing already-available information and where they are discussing their methods/findings, else, they risk presenting a descriptive paper that may not be strongly showcasing their contribution to knowledge.*

Response: Thank you for this feedback. We have divided the Results and Discussion section into two separate sections, making our results more clearly marked. In this way, the results of the research we conducted are more visible.

- Comment 3: *The presentation of the results here is not laid out in a way that a reader can follow in a step wise manner. In modelling specific points in a pathway, it is important for the reader to know what sequence the authors are following in describing their results, especially when it is all tied up in a theory, else the work becomes entirely descriptive and it becomes difficult to differentiate published work from new findings. If it helps, the authors may consider separating the results from the discussion section so that they can explore each section individually and help the reader arrive at a logical conclusion of whether their claims are valid or not.*

Response: Thank you for your suggestion. We have separated the Results and Discussion sections so that the visibility of the results obtained from our research is more emphasized.

Minor points:

- *Line 92-94: Fig 1 is presented as a low resolution image, making it difficult/impossible to review.*

Response: We have revised figures in the paper. We agree with your point and we refined mentioned figure in order to improve its quality and readability.

- *Line 106: It seems intuitive to simple state that “The endpoints of the signaling pathway..”, but the authors may consider stating which pathway they are refereeing to.*

Response: Thank you for pointing this out. As suggested, we have added an explanation that we meant ‘Akt-mediated insulin signaling pathway’ in this sentence.

- *Line 198-207: The effect of lowering mTORC1 activity is described here, but the authors should consider leveraging evidences from literature to bolster their findings.*

Response: Thank you for pointing this out. We have added a piece of information in which we refer to literature results that support the results we obtained. “Similar conclusions can be drawn from the results presented by Rajan et al. (39) and Veilleux et al. (40) which confirms the validity of the method we presented.”

- *Line 214-220: These points here (as in many parts of the write-up) are available in literature and may not be the results for this manuscript. It is therefore important to appropriately reference them, as this is ethically required and further helps the reader to differentiate between the authors results and evidences in literature (even if those evidences were reported by the authors).*

Response: Thank you for raising this issue. We have corrected the indicated fragment to more clearly show the findings of another research team and have cited the work of that team accordingly (lines 226-227).

- *Line 226: “Insulin signaling pathway, disease and treatment strategies”. The authors have majorly discussed mTORC1 activity here, so it is not clear how the subtitle fits the section.*

Response: Thank you for pointing this out. We have decided to remove subsections, because we divided Results and Discussion sections.

- *Line 228-229: “The equations described in the model are based on the mass action law”. Please clarify which model is been referred to; the model used by Sonntag et al. or the model used in this study?*

Response: Thank you for pointing this out. We added an explanation which model is been referred to.

- *Data availability: The authors state that “Yes - all data are fully available without restriction”, is this by request or in a publicly available platform?*

Response: Thank you for your question. The data will be available in the online version of the paper (tables with values of literature concentrations used in the model and reaction equations and kinetic constants used in the model). The source code is freely available for download at https://github.com/UTP-WTIiE/IrsMtorcQueuesSimulation, implemented in C# supported in Linux or MS Windows.

We have also generated a DOI for our GitHub repositorium, which can be accessed here: https://doi.org/10.5281/zenodo.7117138.

We look forward to hearing from you in due time regarding our submission and to respond to any further questions and comments you may have.

Sincerely,

Sylwester Michał Kloska on behalf of all co-authors
