# Supplementary material for: Queueing theory model of mTOR complexes’ impact on Akt-mediated adipocytes response to insulin
Source: PLoS One. 2022 Dec 27;17(12):e0279573. doi: 10.1371/journal.pone.0279573 (PMC9794039; doi:10.1371/journal.pone.0279573)
Supplement: Attachment — Submitted filename: Response to Reviewers.docx [file pone.0279573.s003.docx]

**Dear Dr Irina U Agoulnik**,

Thank you for giving us the opportunity to submit a revised draft of our manuscript titled “Queueing theory model of mTOR complexes’ impact on Akt-mediated cell response to insulin” to *PLOS ONE*. We appreciate the time and effort that you and the reviewers have dedicated to providing your valuable feedback on my manuscript. We are grateful to the reviewers for their insightful comments on my paper. We have been able to incorporate changes to reflect most of the suggestions provided by the reviewers. We have highlighted the changes within the manuscript.

Here is a point-by-point response to the reviewers’ comments and concerns.

**Comments from the Editor**

*Please review your reference list to ensure that it is complete and correct. If you have cited papers that have been retracted, please include the rationale for doing so in the manuscript text, or remove these references and replace them with relevant current references. Any changes to the reference list should be mentioned in the rebuttal letter that accompanies your revised manuscript. If you need to cite a retracted article, indicate the article’s retracted status in the References list and also include a citation and full reference for the retraction notice.*

Response: Thank you for these notifications. We have checked references and we believe there is no retracted paper what we refer to. We have made every effort to ensure that the list of references is complete and meets the requirements of the Journal.

**Comments from Reviewer 1**

- *The authors have done well to respond to the suggestions. They have correctly shown in the methodology that their model and supporting (referenced) experimental result is in* *adipocytes. They may choose to also let this reflect in the title and/or abstract so as to guide readers to the specificity of their work.*

Response: Thank you for your suggestions. We have changed the manuscript title and abstract. They now clearly show that the paper is related to adipocytes response to insulin.

- *Also, appropriate referencing should be made for line 31-36, 63-67 and 121-122.*

Response: Thank you for pointing this out. We have added new references in the mentioned lines.

- *Furthermore, the authors may choose to reword their conclusion as the novelty of their work doesn’t seem to be that “manipulating mTORC1 kinase affects GLUT 4 translocation and glucose entry”, but that* *queuing theory can effectively model the process.*

Response: Thank you for pointing this out. We agree with the comment. The relevant statement has been included in the Conclusions section.

- *Importantly, the authors have alluded to the improvement of queuing theory over ODE’s. are there any drawbacks to modelling with the theory, especially in relation to mTORC1 mediated GLUT 4 translocation?*

Response: The presented model has several limitations. It does not take into account other signaling pathways or individual reactions that are also connected to and influence signaling proteins. This is especially true for the Akt protein, which is the central node in the presented signaling model. Moreover, a model based on literature data will only be as good as the available data. However, we do not question the reliability of other research teams and their published results. Another of the limitations is that in queueing theory, each simulation gives one realization of the stochastic process, while ODE gives an averaged solution. Therefore, a limitation is that depending on the number of cells for which one runs simulations and then averages them, this is how accurate the result will be. Therefore, the model presented here is for averaged results for 50 cells.

We look forward to hearing from you in due time regarding our submission and to respond to any further questions and comments you may have.

Sincerely,

Sylwester Michał Kloska on behalf of all co-authors
